# Laryngeal Langerhans Cell Histiocytosis Presenting with Neck Mass in an Adult Woman

**DOI:** 10.1155/2016/2175856

**Published:** 2016-04-05

**Authors:** Hesam Jahandideh, Yasser Nasoori, Sara Rostami, Mahdi Safdarian

**Affiliations:** ENT and Head & Neck Surgery Research Center, Department of Otolaryngoloy, Head and Neck Surgery, Hazrat Rasool Hospital, Iran University of Medical Sciences, Tehran 11365-3876, Iran

## Abstract

Langerhans cell histiocytosis (LCH) is a very rare condition that commonly affects the head and neck region. There are very few cases of isolated laryngeal involvement by LCH, mostly reported in pediatric patients. Here, we report a case of laryngeal LCH in a 62-year-old woman presenting with a neck mass several weeks ago. The clinical and histopathological findings are reported with a brief discussion about the disease.

## 1. Introduction

Langerhans cell histiocytosis (LCH), formerly known as histiocytosis X, is a rare condition with an unknown etiology, characterized by proliferation of Langerhans cells in different organs [1]. The incidence rate is estimated to be about 1-2 per million individuals [1, 2]. Between different sites of involvement, the head and neck region is affected in almost 90% of the cases. LCH can affect patients of any age; however, it is usually reported in pediatric population [3]. Laryngeal involvement by LCH is extremely rare and very few cases of isolated laryngeal LCH are reported in the literature [4, 5]. Here, we present clinical and histopathological findings of a laryngeal case of LCH in an old female presenting with a neck mass several weeks ago.

## 2. Case Report

A 62-year-old female was admitted to our otolaryngology department with the complaint of hoarseness and a firm swelling right neck mass 3 months ago. In her past medical history, she had hypothyroidism and diabetes insipidus (DI) being treated with levothyroxine tablet 0.1 mg daily and desmopressin spray, one puff every 6 hours, respectively. She was also receiving propranolol tablet 20 mg daily and atorvastatin tablet 20 mg every night. The MRI of pituitary gland reported partially empty sella with a flattened pituitary gland. Her previous medical history was otherwise unremarkable; specifically, there had been no earlier laryngeal disease or signs of upper airway obstruction. She had no history of aspiration or previous intubation. There was no significant finding in her family or habitual history. She was not a smoker and had no history of voice abuse. 

**Figure 1:**
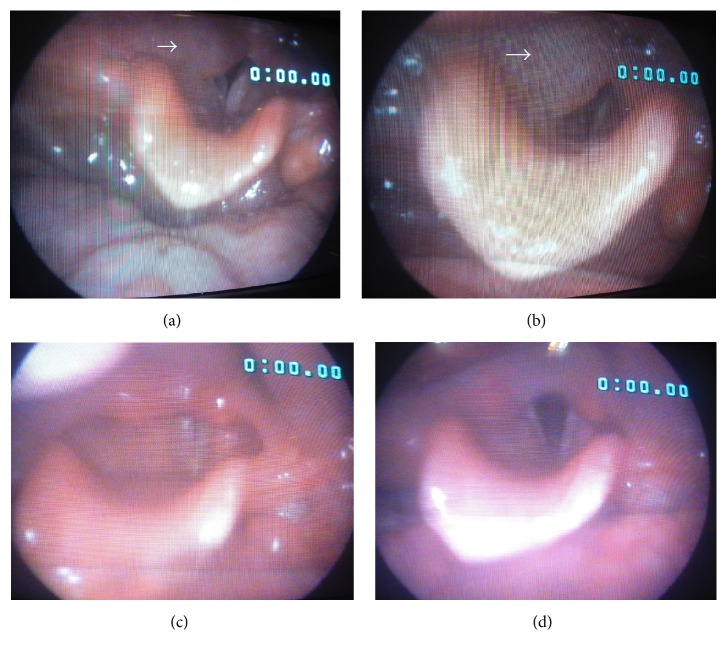
The stroboscopic view of the laryngeal lesion before (upper row: (a), (b)) and after (lower row: (c), (d)) radiotherapy.

**Figure 2:**
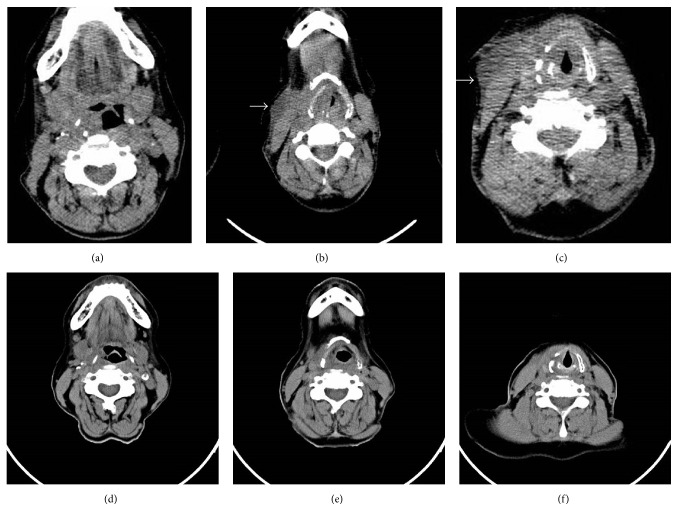
Cervical CT-scan of the laryngeal mass before (upper row: (a), (b), and (c)) and after (lower row: (d), (e), and (f)) radiotherapy.

**Figure 3:**
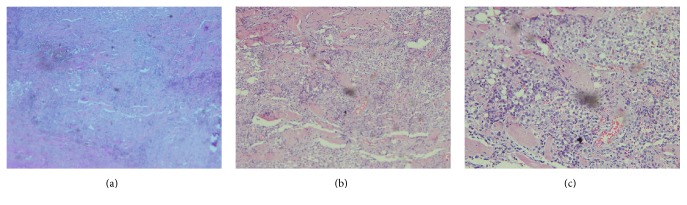
Histological view of the laryngeal lesion (magnification: (a) ×10, (b) ×100, and (c) ×1000).

Physical examination showed a 5*∗*5 cm supraglottic mass with extension to the pharynx. Direct laryngoscopy revealed a hypopharyngeal mass with normal epithelium, while the movement of true vocal cords could not be assessed. The physical examination of other organs was otherwise normal. The fine needle aspiration (FNA) cytology from the right neck laryngeal mass showed hypercellular smears composed of some dissociated atypical cells with large pleomorphic nuclei and high nucleus-to-cytoplasm (N/C) ratio mixed with acute inflammatory cells in a necrotic background. Reactive lymphoid tissue lined by partially atrophied squamous epithelium was reported in addition to some atypical large lymphocytes invading epithelium, suggesting lymphoepithelial lesion with extensive necrosis.  Figure 1 shows the stroboscopic view of the lesion and cervical CT-scan of the laryngeal mass is shown in Figures 2(a)–2(c). The incisional biopsy of the right cervical mass was done with a macroscopic feature of a creamy soft tissue. Sections showed neoplastic tissue composed of large mononuclear and few multinuclear cells sheets admixed with eosinophils, which infiltrated muscle bundles on microscopic evaluations. Tumor cells had irregular nuclei with prominent grooves, folds, and inconspicuous nucleoli.  Figure 3 shows the histological view of the laryngeal lesion. The diagnosis was suggested to be LCH (histiocytosis X). The tumor markers CD1a and S100 were positive in the immunohistochemical (IHC) staining. Based on the clinical and cytomorphological findings, a diagnosis of laryngeal LCH was made for the patient. In order to rule out the other potentially involved sites, a whole body bone scan and a chest CT-scan were done for the patient, which were both negative for an extra involvement site. Although surgical excision is considered as the standard treatment, anticipated morbidity, due to extensive surgical procedure of laryngopharyngectomy, convinced us to choose low-dose radiation. The patient had 10 sessions of cervical radiotherapy with a dose of 2000 cGy at each session. This protocol was well tolerated with a good response to treatment (Figures 2(d)–2(f)).

## 3. Discussion

LCH is a rare disease that occurs mainly in children, and males are more often affected than females [4]. The clinical presentation of LCH is highly variable in relation to the patient's age and includes swelling (64%), pain (9%), or both (18%). The skull (61%) and the orbit (24%) are the most common location for isolated bone lesions in the head and neck region [5]. Definitive diagnosis depends on the identification of characteristic IHC or ultrastructural features of the biopsy specimen [6].

Langerhans cells show strong positivity by IHC studies for S100 protein and CD1a [7]. While there is no established standardized treatment protocol for LCH, prognosis in adults is generally good [1] and is highly dependent on the age and number of systems involved [8]. In a retrospective study of twenty-two patients with LCH that primarily affected head and neck sites between 1986 and 2004, seventeen (77%) patients had head and neck involvement; 14 (64%) of these patients had primary head and neck LCH. Overall outcomes were good with 10 of 14 patients without disease at the last follow-up; however, recurrence was common and involved 50% of the patients [9]. A summary of studies reporting pediatric LCH by Buchmann et al. showed that the most common site of involvement of LCH was the head and neck region [9].

In a retrospective study to appreciate the several head and neck manifestations of LCH in children and their multidisciplinary management and outcome, 31 (73.8%) of the 42 patients presented with head and neck localization, 10 of them had an exclusive head and neck presentation. All treatments delivered to patients were well tolerated and the evolution was good [3]. Although head and neck region is the most common reported site of involvement by LCH, there are very few cases of laryngeal LCH in the literature and are usually reported in pediatric patients. Booth and Thomas described the first case of isolated laryngeal LCH in 1970 [7, 8]. Duynstee et al. reported a case of isolated LCH in a 9-year-old girl who was presented with subglottic stenosis [4].

Treatment of LCH depends highly on the involved organs and includes radiation therapy, chemotherapy, and surgery. In a systematic review by Bezdjian et al. to provide a treatment algorithm for isolated LCH bone lesions in pediatric patients, the most frequently documented treatment option was reported to be resection, followed by observation, chemotherapy, and intralesional steroid injection [5].

Radiotherapy for LCH has been reported to have high rates of local control and symptomatic improvement. However, there is evidence of short-term and long-term morbidity when children are treated with low-dose irradiation. Lallemant et al. recommend wait and see policy or chemotherapy instead of aggressive local treatments including surgery or radiotherapy as the favorable therapeutic approaches [10].

Considering the multisystem expression of the disease, a thorough screening of additional potentially involved sites, including lung, bone, teeth, skin, mucosa, is highly recommended. BRAF mutation testing which is a powerful molecular marker for papillary thyroid carcinoma, cutaneous malignant melanoma, and hairy cell leukemia is also recommended. Cancers with a BRAF mutation are generally more aggressive than their counterparts without the mutation [11]. Here, we reported a case of LCH with isolated laryngeal involvement in a female adult patient. The skull base and the pituitary glands, which are the most reported sites of involvement by LCH in the head and neck region, were intact in our patient, based on the MRI findings, and the diagnosis was confirmed by IHC anti-S100 and CD1a positivity.

Interestingly, our patient had been under treatment for DI before the diagnosis of LCH, which is the most common manifestation of central nervous system (CNS) involvement in LCH [12]. Patients with LCH involving the head and neck region are reported to have about a 40% lifetime chance of developing DI [12]. The clinical and biochemical diagnosis of DI is sometimes supported by the absence of the posterior pituitary bright signal on magnetic resonance images. As in our patient, the MRI showed a partially empty sella with a flattened pituitary gland. In a study on 1,741 patients with LCH to define the population at risk for DI, 12% of the patients were reported to have DI, while DI was present at diagnosis of LCH in 6% of the patients [13]. Our patient was also a known case of DI at the time of LCH diagnosis. The risk of developing DI was reported to be 20% at 15 years after diagnosis in the aforementioned study. The authors concluded that patients with multisystem disease and craniofacial involvement at diagnosis, in particular the “ear,” “eye,” and the “oral region,” carry a significantly increased risk to develop DI during their course. This risk is augmented when the disease remains active for a longer period or reactivates [13]. Since LCH is the most common systemic disease that may cause DI, special focus should be paid to the identification of LCH lesions. Our patient had previously developed DI, which is a part of the natural history of LCH, but she had never been carefully evaluated for LCH due to the lack of clinical suspicious. The recent presentation of LCH as a laryngeal mass however uncovered the underlying cause for her DI.

## 4. Conclusion

LCH is a very rare entity usually affecting pediatric population. However, the isolated laryngeal presentation of LCH in an adult patient would be much rarer. Otolaryngologists need to be familiar with its presentation, workup, and treatment, due to the frequent head and neck involvement of this disease. Paying more attention to LCH is recommended in idiopathic DI patients since it is the most common CNS involvement in LCH.
